# Assembly of Copper Phthalocyanine on TiO_2_ Nanorod Arrays as Co-catalyst for Enhanced Photoelectrochemical Water Splitting

**DOI:** 10.3389/fchem.2019.00334

**Published:** 2019-05-14

**Authors:** Yuangang Li, Mengru Yang, Zimin Tian, Ningdan Luo, Yan Li, Haohao Zhang, Anning Zhou, Shanxin Xiong

**Affiliations:** ^1^College of Chemistry and Chemical Engineering, Xi'an University of Science and Technology, Xi'an, China; ^2^Key Laboratory of Coal Resources Exploration and Comprehensive Utilization, Xi'an, China; ^3^Key Laboratory of Synthetic and Natural Functional Molecule Chemistry of the Ministry of Education, College of Chemistry and Materials Science, Northwest University, Xi'an, China

**Keywords:** self-assembly, copper phthalocyanine, TiO_2_, water oxidation, surficial naostructutre

## Abstract

A photoelectrochemical device was achieved by interfacial self-assembly of macrocyclic π-conjugated copper phthalocyanine (CuPc) on surface of TiO_2_ nanorod arrays (NRs). The photocurrent density of the elegant TiO_2_@CuPc NRs photoanode reaches 2.40 mA/cm^2^ at 1.23 V vs. RHE under the illumination of 100 mW/cm^2^ from AM 1.5G sun simulator, which is 2.4 times higher than that of the pure TiO_2_. At the same time, the photoelectrochemical device constructed through this strategy has good stability and the photocurrent density remain almost no decline after 8 h of continuous operation. The Mott-Schottky and LSV curves demonstrate that CuPc act as a co-catalyst for water oxidation and a possible mechanism is proposed for water oxidation based on careful analysis of the detailed results. The holes from VB of TiO_2_ photogenerated by electrons exciting are consumed by a process in which Cu^2+^ is oxidized to Cu^3+^ and Cu^4+^, and then oxidize water to produce oxygen. CuPc species is considered to be a fast redox mediator to reduce the activation energy of water oxidation in and effectively promote charge separation.

## Introduction

Photoelectrochemical (PEC) water splitting is considered as one of the most promising technologies to address the challenges of impending worldwide energy consuming and associated climate change resulting from combustion of fossil fuels (Du and Eisenberg, 2012; Wang et al., 2016; Seh et al., 2017). Through this promising strategy, hydrogen could be produced as a clean fuel by sun light photolysis of water under the help of a bias potential (Ferreira et al., 2004; Gibson et al., 2017; Li et al., 2017). However, the sluggish kinetics of the two half-reactions of water splitting, especially the more sluggish four-electron oxygen evolution reaction, always leads to poor performance of the PEC device and relatively low energy efficiency (Haumann et al., 2005; Bessel et al., 2010; Fang et al., 2017). Therefore, many research works in the past have still focused on the design and improving the performance of photo anode on which water oxidation occurred, although the more desired hydrogen evolution reaction occurred on the photo cathode (Osterloh, 2013; Meyer et al., 2017). In order to achieve PEC device with relatively high performance, many strategies have been employed. One of the effective strategies is adopting photo electrode of vertically aligned arrays (Lee and Chen, 2014) compositing of low dimensional nanostructures such as nanorods (Bin and Aydil, 2009; Liu B. et al., 2013; Li et al., 2016b; Tang et al., 2017), nanowires (Hang et al., 2001; Li et al., 2016a; Zhang et al., 2016; Jeong et al., 2018; Yao et al., 2018), and nanosheets (Zhu et al., 2011; Yang et al., 2012; Du et al., 2013; Zhang et al., 2014; Zhang R. et al., 2018; Shi et al., 2018; Zhao et al., 2018a) because this kind of interfacial nanostructures can decouple the length scales of charge diffusion and light absorption, at the same time offering sufficient surface area for the photogenerated electrons or holes to diffuse onto the interface of electrolyte and electrode (Xiao et al., 2015). TiO_2_ nanorod arrays (Akira and Kenichi, 1972; Liu L. et al., 2013; Li et al., 2015, 2016c) had been applied extensively in PEC water splitting as for its low cost, non-toxic, and stable performance.

Another effective way is selecting a suitable co-catalyst to improve the photo electrochemical performance and water oxidation activity in the process of assembling photo electrochemical device (Ran et al., 2014; Ding et al., 2017; Zhang Y. et al., 2018). In the process of PEC water splitting, co-catalysts play three pivotal roles for improving the reliability and activity of semiconductor: (i) co-catalysts could reduce the over-potential (Artero et al., 2011) or activation energy for oxygen production reactions on the surface of semiconductors; (ii) co-catalysts are capable of slowing electron-hole recombination at the interface between co-catalyst and semiconductor; (iii) co-catalysts could improve the stability of semiconductor photo electrode and suppress the photo-corrosion. Over the past few years, the advances of electrode manufacturing technology and materials science have greatly expanded in O_2_ evolution (Li et al., 2012; Nepal and Das, 2013; Lauinger et al., 2015; Gong et al., 2016). Many kinds of novel co-catalysts and interlayers were successfully loaded on various photoelectrodes (Long et al., 2018; Zhao et al., 2018b; Yin et al., 2019). Thus far, noble-metal oxides (IrO_2_ or RuO_2_) act as the best accepted O_2_-evolution co-catalysts, which have lower over-potential for oxygen evolution in acidic conditions (Junya et al., 2005; Blakemore et al., 2010; Cherevko et al., 2015).

With the exception of rare and precious metals, some noble-metal-free and low-cost transitional metals, such as Co (Youn et al., 2015; Zhang et al., 2017), Ni (Yoon et al., 2015) and Fe (Youn et al., 2015) have also been used as co-catalysts in PEC O_2_ production. Although copper (Zhou et al., 2015) based catalyst often has been applied for H_2_ evolution (Kumar et al., 2016), hardly for O_2_ production (Terao et al., 2016; Chauhan et al., 2017), in Lu group (Lu et al., 2016), Cu(II) aliphatic diamine complexes was immobilized on ITO as heterogeneous water oxidation catalysts. In Su (Su et al., 2015) and Zhang (Zhang et al., 2013) group, copper complexes were synthesized as homogeneous O_2_ production catalysts. Copper phthalocyanine is a kind of organic heterocyclic compounds, containing large ring Π conjugated structure with good stability and effective property (Jiang et al., 2017).

It has been proven that supramolecular self-assembly of pi-conjugated molecules was a good strategy to fabricate various functional organic-based nanostructured materials, which aims to manufacturing sophisticated organized molecular aggregate, through various non-covalent interactions, including hydrophobic interactions, π-π interactions, electrostatic interactions, etc. (Chen et al., 2008; Zhang et al., 2009; Qiu et al., 2010; Guo et al., 2011, 2012). Although, supramolecular assembled nanostructures (Wang et al., 2014; Liu et al., 2015; Geng et al., 2018) based on pi-conjugated phthalocyanine derivatives have been reported to be effective in the field of laser printing, xerography, organic solar cells, etc., it is very rare to apply this kind of strategy for constructing PEC device. Herein, we adopted an electro-induced surficial assembly method to construct PEC device with enhanced performance. We employ copper phthalocyanine (CuPc) as an O_2_ evolution co-catalyst and TiO_2_ nanorod arrays (TNRAs) as semicondustor for light harvesting. The obtained surficial nanostructured assembly could be used as photo anode for efficient PEC water splitting. The photocurrent density of the elegant CuPc assembled TiO_2_ nanorod arrays (CTNRAs) photoanode reaches 2.4 mA/cm^2^ at 1.23 V vs. RHE, which is more than 2 folders higher than that of the pristine TNRAs and the stability of the PEC device is also very good. There have no obvious performance decline after 8 h continuous operation. A possible mechanism for PEC water oxidation on CTNRAs was proposed based on detailed experiments.

## Experimental Section

### Sample Preparation

#### Materials

All chemicals were analytical grade and used without further treatment. Fluorine-doped tin oxide (FTO) substrates (14 Ω/square) were obtained from Huanan Xiangcheng Technology Co., Ltd. Copper phthalocyanine (C_32_H_16_CuN_8_), tetrabutyl titanate (C_16_H_36_O_4_Ti), Trifluoroacetic acid (CF_3_COOH), and methanol (CH_3_OH) were purchased from Aladdin Chemical Reagent Co. Ltd. In addition, acetone (C_3_H_6_O), hydrochloric acid (HCl), and absolute ethanol (C_2_H_5_OH) were bought from Sinopharm Chemical Reagent Co. Ltd. Ultrapure water was used in the experiments.

##### Preparation of FTO@TiO_2_ (TNRAs) and FTO@TiO_2_@CuPc (CTNRAs)

*Preparation of TNRAs* TiO_2_ nanorod arrays were prepared by hydrothermal method in autoclave (Bin and Aydil, 2009). Firstly, FTO were cleaned ultrasonically three times in solvents of acetone, ethanol and deionized water alternatively 15 min per step, and were dried in vacuum dryer at 60°C. Next, 0.4 mL tetrabutyl titanate were added drop wise into uniform mixed solution of 12 mL ultrapure water and 12 mL hydrochloric acid (mass fraction 36.5–38 %). When the mixture solution of precursor was clarified, we transferred it into 100 mL of Teflon-lined stainless steel autoclave. The conductive surface of FTO was put downwards at a slight angle with the inner wall of autoclave. Then, the autoclave was kept in an oven of 150°C for 15 h and cooled to 25°C. Then the FTO with nanorod arrays of TiO_2_ were taken out and dried. Finally, the TiO_2_ nanorod arrays were annealed at 450°C for 1 h.

**Figure 1:**
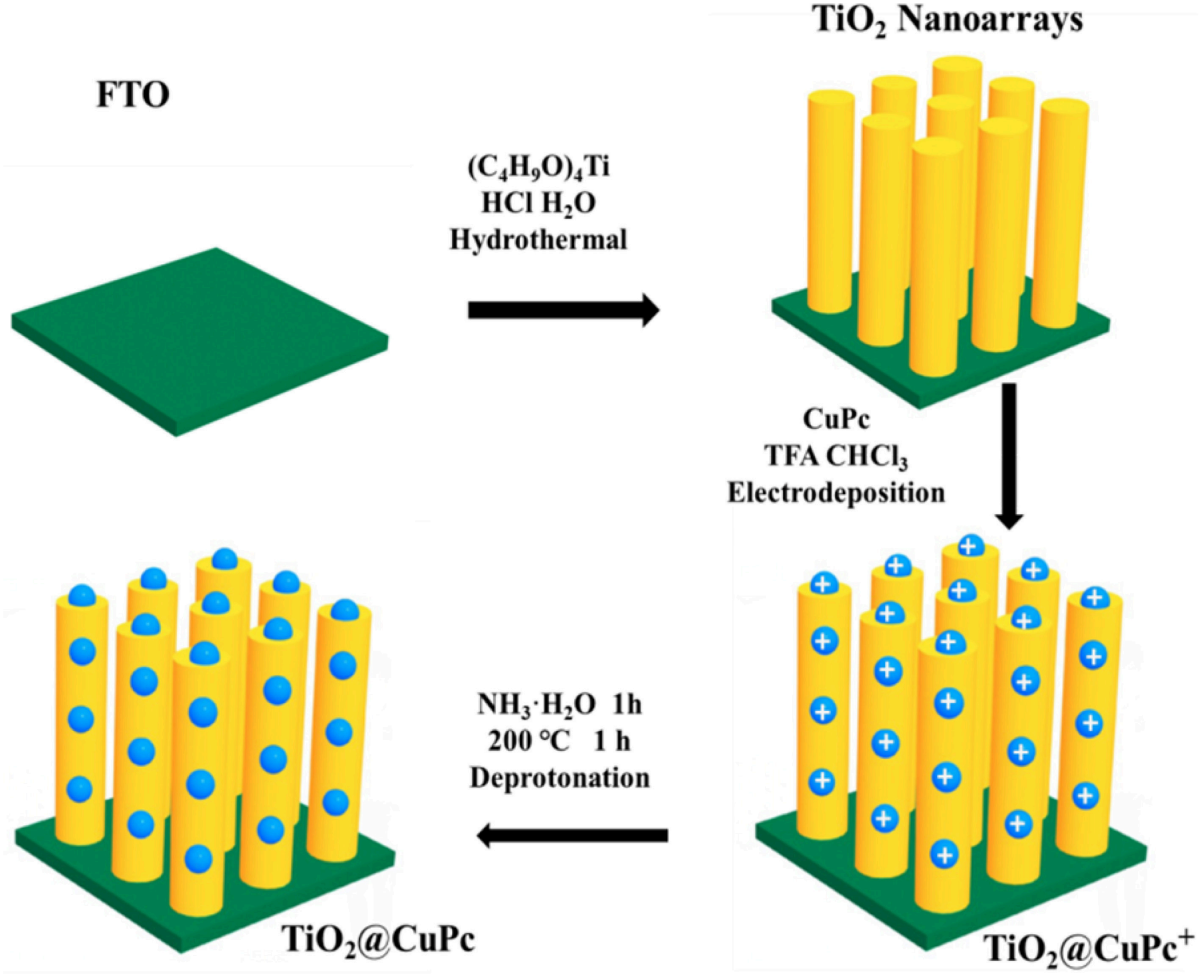
Schematic illustration for the preparation of CTNRAs.

*Preparation of CTNRAs* For synthesis of CTNRAs, 3 μmol CuPc were dissolved in 5 mmol trifluoroacetic acid, which mixed with 50 mL chloroform as electroplate solution (Ogunsipe and Nyokong, 2004). The mono-protonated [CuPc·H]^+^ (Figure S1A) and di-protonated [CuPc·H_2_]^2+^ (Figures S1B,C) forms of copper phthalocyanine (CuPc) were obtained by TFA (Su et al., 2009). The CTNRAs was fabricated by the electrophoretic deposition (EPD) method from the protonated CuPc dissolved in chloroform containing TFA. Then The CuPc^+^ ([CuPc·H]^+^ and [CuPc·H_2_]^2+^) was loaded on TNRAs by electrodeposition of 30, 60, 90, 120, and 150 s. Next CuPc^+^ were deprotonated by dipping the TNRAs substrate in 1 M ammonia for 1 h and drying for 1 h at 200°C and CuPc was successfully covered onto TiO_2_ nanorod arrays (Figure 1). FTO@CuPc was prepared in the same way.

### Materials Characterizations

XRD were characterized by a Shimadzu 7000S X-ray diffract meter with Cu-Kα radiation and operating in a 2θ range of 20–70° at a scan rate of 5° per minute. XPS patterns of the as-prepared samples were carried on a Kratos Axis Ultra DLD system with an Al Kα X-ray source (hν = 1486.69 eV). The morphologies, EDS mapping analyse and high-resolution images were studied by a field emission Tecnai G2 F20 transmission electron microscopy with an acceleration voltage of 200 kV. SEM images were achieved using a Hitachi S-4800 field-emission scanning electron microscopy. Raman spectra were investigated on a WITec Alpha 300 R Confocal Raman Spectrometer, which has an excitation wavelength of 532 nm at the room temperature. The UV-vis spectra were characterized by a Shimadzu UV-2550 UV-Vis spectrophotometer using BaSO_4_ as the reference. The linear sweep voltammetry (LSV), electrochemical impedance spectroscopy (EIS) and cycle voltammetry (CV) were conducted using an electrochemical workstation (CHI 660E) in a three-electrode system. The incident photon-to-current conversion efficiency (IPCE) was measured by the same workstation and a Xenon lamp (300 W) coupled with a monochromator. Inductively coupled plasma (ICP) measurement was carried on Varian 715-ES.

### PEC Measurements

PEC water oxidation was investigated using a three-electrode potential station (CHI 660 E, China) with a saturated Ag/AgCl (in 3 M KCl) as reference electrode and a platinum wire as counter electrode. The schematic illustration for the geometry and design of PEC reactor was shown in Figure S16. Under AM 1.5G simulated solar light illumination (100 mW/cm^2^) from a Xe lamp (300 W), the as-prepared working electrodes exhibit photoelectron activity for water splitting. 0.1 M sodium sulfate solution (pH ≈ 6.8) was used as electrolyte with 30 min N_2_ bubbling. LSV was conducted at a scan rate of 10 mV/s. The potential of the working electrodes (vs. Ag/AgCl) can be converted to the reversible hydrogen electrode (RHE) by the Nernst equation:

$$E_{RHE}=E_{Ag/AgCl}+0.0591pH+0.19742$$

## Result and Discussion

**Figure 2:**
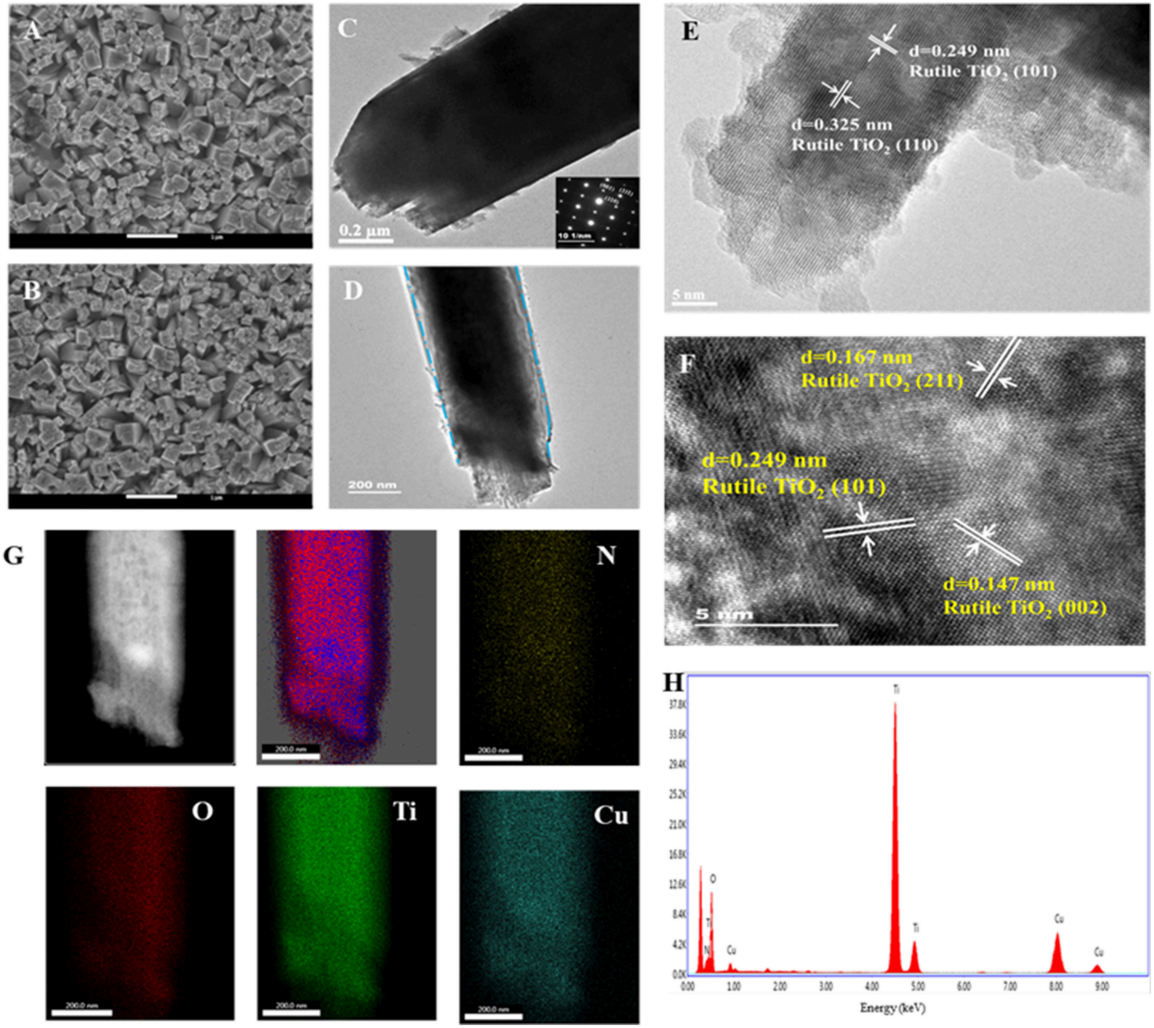
Typical top-view SEM images of **(A)** pure TNRAs and **(B)** the CTNRAs; TEM images of **(C)** pure TNRAs and **(D)** the CTNRAs; HR-TEM images of **(E)** pure TNRAs and **(F)** the CTNRAs; **(G)** EDX elemental mapping of N, O, Ti, and Cu; **(H)** EDX spectrum of the CTNRAs.

TNRAs on FTO were prepared in an autoclave by a hydrothermal method. The obtained TNRAs are almost vertically grown on the FTO substrate, with an average diameter and length of ~500 nm and ~3 μm, respectively (Figure 2A and Figure S4A). Each nanorod consisted of several smaller rods as shown in Figure S1C. Transmission electron microscopy picture from a single nanorod (Figure 2C, and Figures S2A,B) shown that the diameter observed is about 600~800 nm which is in accordance with SEM observation. High-resolution transmission electron microscopy (HR-TEM) of TNRAs showed that two kinds of lattice fringes could be clearly observed and the distances between adjacent lattice fringes were about 0.325 and 0.249 nm (Figure 2E), respectively. After careful comparison with the results of literature we attribute them as lattice fringes from the crystal lattice of (110) and (101) of rutile TiO_2_, respectively, illustrating that the obtained TNRAs is rutile TiO_2_ and grow along with the direction of (001) lattice (Wang et al., 2011).

The CTNRAs were fabricated through a simple electrodeposition method, as illustrated in Figure 1. The SEM images of CTNRAs were almost the same as pure TNRAs (Figure 2B and Figures S3C, S4B), which indicate the process of the CuPc deposition does not damage the structure of the pristine TNRAs. But from Figure 2D and Figure S3 we can observe that the surface of TiO_2_ was more rough compared with that of TNRAs. The HR-TEM of CTNRAs reveals a single crystalline structure in Figure 2F. Lattice fringes of 0.147 nm (002), 0.167 nm (211), and 0.249 nm (101) belong to rutile TiO_2_ and which is consistent with that of pure TNRAs. Although the lattice structure of CuPc was not found, the characteristic elements of nitrogen and copper of CuPc could be seen in energy dispersive X-ray elemental mapping (Figures 2G,H). From the elemental mapping images of CTNRAs as shown in Figure 2G, it is observed that the copper and nitrogen elements from CuPc distribute as uniformly as the elements of oxygen and titanium from TiO_2_, which means that CuPc was successfully deposited on the surface of TNRAs and the deposited CuPc distribute uniformly on the whole surface of TiO_2_ nanorod.

**Figure 3:**
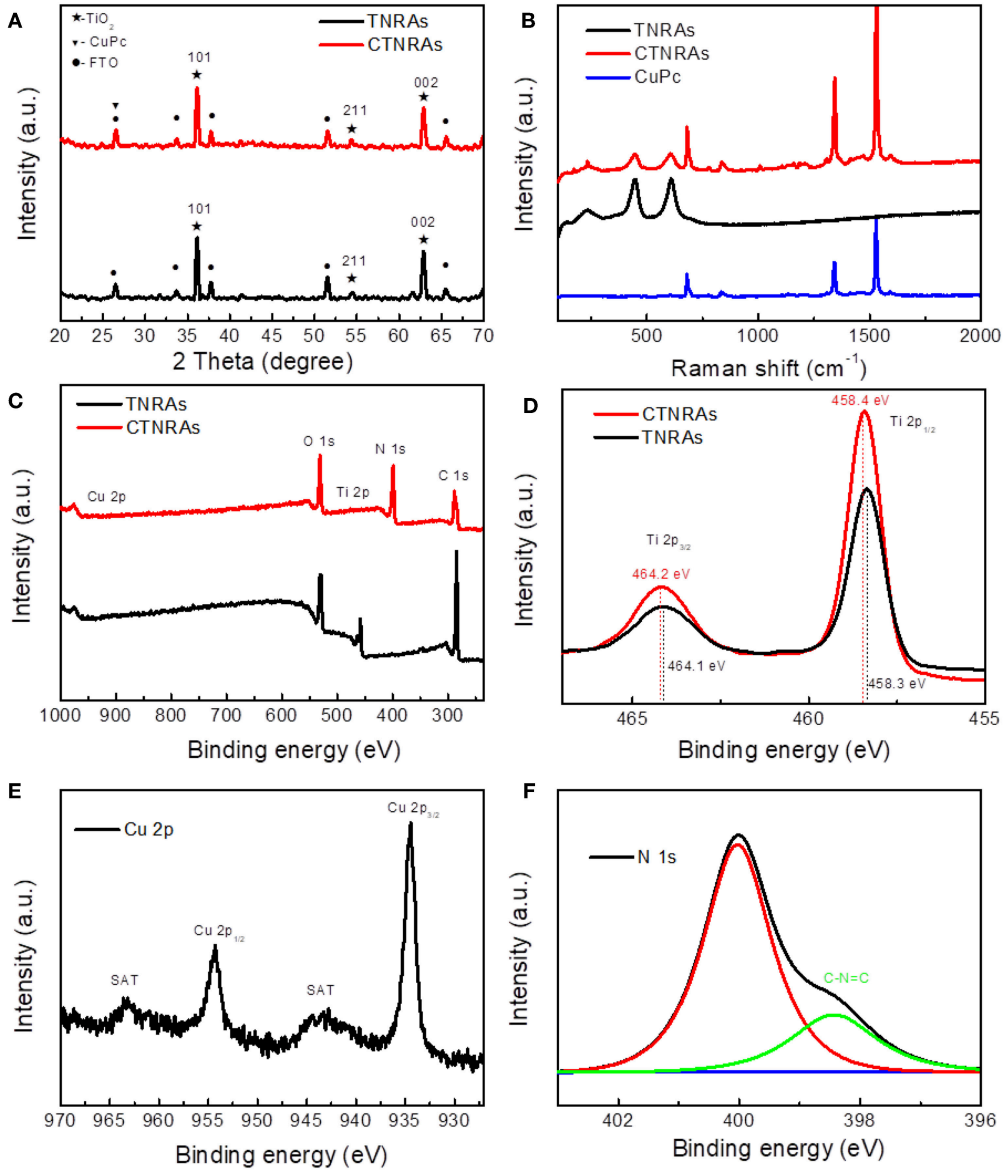
XRD patterns **(A)**, Raman shift spectra **(B)** of pure TNRAs, CuPc and CTNRAs, XPS survey **(C)** of pristine TNRAs and CTNRAs; High resolution XPS spectra of Ti 2p **(D)**, Cu 2p **(E)**, and N 1s **(F)**.

X-ray diffraction (XRD) curves (Figure 3A) indicate that both TNRAs and CTNRAs can be ascribed to the tetragonal TiO_2_ with the rutile phase (JCPDS 21-1276). As for pristine TiO_2_, the main distinct diffraction peaks at 36.3°, 54.5°, and 62.6° can be well attributed to (101), (211), and (002) crystal planes, respectively. The XRD spectra are consistent with HR-TEM patterns. The other distinct diffraction peaks from TNRAs located at 2θ values of 27.2°, 33.7°, 38.1°, 57.1°, and 66.2 ° are attributed to SnO_2_ from FTO substrate. No apparent peaks of CuPc can be observed in CTNRAs samples due to the low loading of CuPc, or the amorphous state of CuPc from electrodeposition method.

Raman spectroscopy measurements were employed to further observe the existence of CuPc on the surface of CTNRAs. The data shown in Figure 3B clearly proved that CuPc was successfully deposited on TNRAs to form CTNRAs. The spectrum of pristine TNRAs show four characteristic peaks at 143, 448, 608, and 238 cm^−1^, respectively, matching well with the B1g, Eg, A1g of rutile TiO_2_ and lattice distortion or multi spectral of rutile TiO_2_. In contrast, the characteristic Raman peaks located at 1,535, 1,338, and 685 cm^−1^ are observed in the CuPc samples (Ludemann et al., 2011), which could be accordance with the C-N stretching vibration on pyrrole bonded to central copper ion, C-N stretching vibration peak on heterocyclic ring and vibration of large phthalocyanine ring, resprctively. As for the sample of CTNRAs all characteristic peaks of CuPc and rutile TiO_2_ could be seen with a slightly shift, which further elucidate the existence of CuPc on CTNRAs. Optical properties of CTNRAs, CuPc, and TNRAs were measured by UV-Vis absorption measurement (Figures S6, S7). Owing to the broad band gap (3.0 eV), TiO_2_ only reveal a strong absorption at wavelength shorter than 410 nm. CuPc display strong absorption bands in the range of 500–800 nm. In comparison, TiO_2_@CuPc sample exhibit a strong absorption shorter than 410 nm, and two very weak absorption peaks at about 550 and 650 nm which results from CuPc due to the lower deposition amount.

In order to further confirm the existence of CuPc in TNRAs and understand the surface chemical state of the sample, we have employed the X-ray photoelectron spectroscopy (XPS) measurements. Obvious photoelectron peaks of C, N, Cu, O, and Ti elements are observed in the XPS survey spectrum (Figure 3C) of CTNRAs. In compare with pristine TNRAs, TCNRAs contain not only Ti, O and C elements from TNRAs, but also Cu and N elements from CuPc. The high resolution Ti 2p XPS spectrum (Figure 3D) shows two different peaks at 462.4 eV and 458.4 eV, which can be ascribed to the characteristic of Ti 2p_1/2_ and Ti 2p_2/3_ deriving from Ti^4+^, respectively. Furthermore, the O 1s spectra (Figure S8) of TNRAs and CTNRAs can be factored into two peaks at about 529.6 and 530.1 eV. The peak 1 at about 530.1 eV is ascribed to surface Ti-OH species, while peak 2 is assigned to lattice oxygen in O-Ti^4+^. Compared to pure TiO_2_, the deposition of CuPc reduces the area ratio of peak 1 to peak 2, maybe indicating the surface of TiO_2_ was covered by CuPc, so that it adsorb less amount of OH groups. It can be seen from Figure 3E that the high resolution XPS spectrum of Cu 2p in CTNRAs has two strong peaks at 954.3 and 934.5 eV, which are the Cu 2p_1/2_ and Cu 2p_2/3_, respectively. The binding energy of the Cu 2p2/3 is 934.5 eV, indicating that the Cu atom in the CTNRAs exists in the Cu (II) state (Zheng et al., 2004). This data can be explained by the chemical structure of the CuPc molecule, in which the copper exists in the bivalent form. In addition, the satellite peak of the Cu(II) state can be seen, which imply that the CuPc molecular plane contains 3dx^2^-y^2^ orbitals. From the fine spectrum of N 1s (Figure 3F), we can see two peaks located at 400.1 eV and 398.6 eV. N atoms have two chemical environments in CuPc, one is C-N = C bond forming by 4 C atoms with 2 N atoms which located in 398.6 eV, the other is the 4 N atoms coordination bonding with the Cu atom, the signal of which located at 400.1 eV. As for high resolution XPS spectrum of C 1s (Figure S9), there are two types of carbon atoms in the CTNRAs 8 C atoms are bonded to 2 N atoms forming N-C = N bond at 289.4 eV; the remaining 24 C atoms have aromatic hydrocarbon properties which is located at 285.0 eV. With decided contrast, the XPS spectrum of pure TNRAs only have a C 1S signal at 284.8 eV, which can be attributed to the absorbed contaminants. Combined with the results of XRD analysis, Raman spectra and UV-Vis absorption, we concluded that CuPc indeed assembled onto the surface of TNRAs to form a uniform coverage through electro-induced assembly. As far as the assembled pattern of CuPc on the surface of TiO_2_ nanord was concerned, we inferred that the plane CuPc molecules adopt a flattened pattern rather than a standing pattern on the surface of TiO_2_ nanorod through electrostatic self-assembly because CuPc have a plane structure and the positive charge located near the center of the plane, so the flattened pattern is more stable than the standing pattern, which will be proved by the good stability of obtained device for PEC operation.

**Figure 4:**
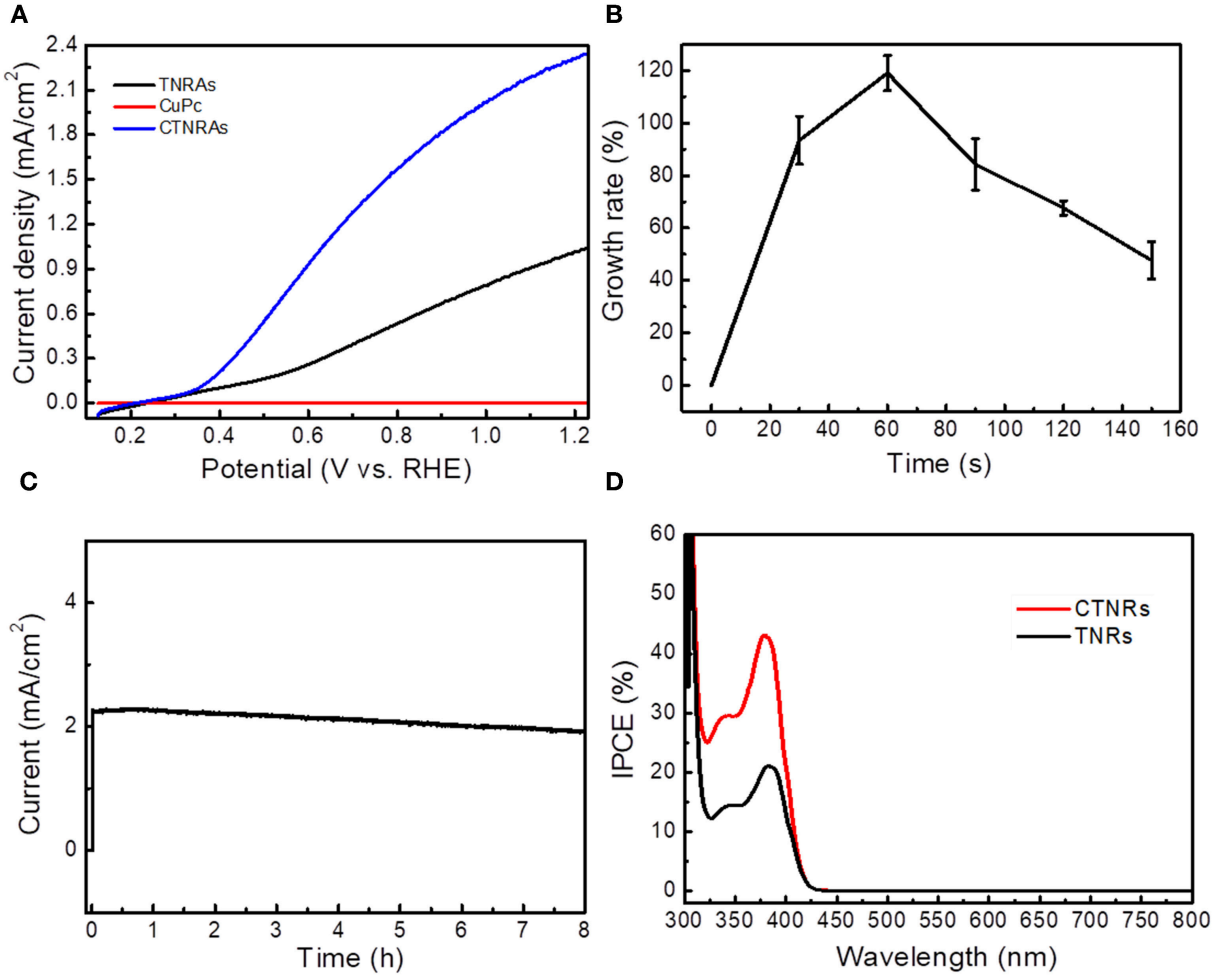
**(A)** Photocurrent density vs. potential curves for the CTNRAs, pristine TNRAs, and CuPc under AM 1.5G (100 mW/cm^2^); **(B)** Growth rate of photocurrent density-potential of CTNRAs with various deposition time; **(C)** Chronoamperometry (i-t) of CTNRAs with a three-electrode system at 1.23 V_RHE_ for 8 h. **(D)** IPCE pectrum of TNRAs and CTNRAs.

The linear sweep voltammetry (LSV) curves showed in Figure 4A reveals the photoelectrochemical (PEC) activities of the as-prepared samples. Under 100 mW/cm^2^ irradiation by the simulated solar light (AM 1.5G), transient photocurrent responses of pristine TNRAs is 1.01 mA/cm^2^ at 1.23 V (vs. RHE), while pure CuPc almost have no PEC response. After coating with CuPc, the photocurrent density of CTNRAs showed dramatically increase. With the electrodeposition time prolonged, the photocurrent density increased first and then decreased (Figure S15). The optimized electrodeposition time is 60 s, the photocurrent density reaches 2.40 mA/cm^2^ at 1.23 V (vs. RHE), which was 2.40 times as that of pure TNRAs. Then, the photocurrent response was decrease when electrodeposition time longer than 60 s. The photocurrent density of CTNRAs was 1.80, 1.60, and 1.30 mA/cm^2^, when the electrodeposition time was 90, 120, and 150 s, respectively.

To more intuitive observe the relationship between the photocurrent density enhancement and the deposition time of the CTNRAs, the curve of growth rate of photocurrent density vs. deposition time was ploted in Figure 4B. Growth rate can be expressed concretely as:

$$Growth\;ratio=\frac{I_{CTNRAs}-I_{TNRAs}}{I_{TNRAs}}\times 100\%$$

Where I_CTNRAs_ and I_TNRAs_ were the photocurret density of CTNRAs and TNRAs, respectively. The plot was shown as a single peak curve that the growth ratio increased rapidly as the deposition time < 60 s and reaches the maximum average growth rate of about 120% at 60 s. When the time was prolonged further, the growth ratio declined with deposition time. ICP-MS measurements was carried out to estimate the loaded amount of CuPc on the surface of TNRAs, when the electrodeposition time was 30, 60, 90, 120, and 150 s, the anchoring mass of CuPc was 0.024, 0.047, 0.069, 0.089, and 0.112 μmol on per square centimeter TiO_2_, respectively. With the amount of CuPc increased, the photocurrent density increased first and then decreased. And the fine XPS spectrum of Cu_2p_ and Ti_2p_ in the optimized CTNRAs sample was calculated carefully to estimate the surface atom ratio of Ti and Cu (Figure S10), the results illustrated that the surface atom ratio of Ti and Cu from the optimized sample was about 30:1.

The stability of the optimized CTNRAs was detected by photocurrent-time (I-t) measurement at 1.23 V vs. RHE under continuous illumination (100 mW/cm^2^, AM 1.5G) for 8 h (Figure 4C). Starting without illumination, the photocurrent density is almost zero. When illumination was opened, the photocurrent density reached 2.40 mA/cm^2^. The value of photocurrent density stays almost no decline in the long 8 h operation which means that CTNRAs have a good stability for PEC water oxidation. To observe the composition and morphology change of the device during the process of PEC operation, SEM, XPS and ICP-MS measurements were conducted. The results shown that: (1) the morphology of the electrode after operation is almost the same as that before operation (Figure S5); (2) XPS measurement (Figures S11, S12) confirmed the exist of CuPc on the sample after operation, but the ICP measurement show that the loaden amount of CuPc is declined compared to the sample before operation. This is probably due to the slight leakage of CuPC during the PEC process.

Incident photon-to-current conversion efficiency (IPCE) was measured at 1.23 V vs. RHE, to further elucidate the enhanced PEC oxygen production performance of CTNRAs. IPCE (Cho et al., 2011) can be calculate concretely as:

$$IPCE=\frac{\left(1240\times J\right)}{(\lambda \times I)}\times 100\%$$

Where λ and I are the incident light wavelength and the power density for each wavelength, respectively, and J is the photocurrent density produced by excited electrons. As shown in Figure 4D, the IPCE of CTNRAs is higher than that of pristine TNRAs in the whole spectrum, which indicates that the photogenerated electron-hole pairs are effectively separated in the CTNRAs. The IPCE value was reached 45% at 380 nm, but the IPCE of pure TNRAs is only 21%. The IPCE value can be improved by enhancing the charge injection efficiency, efficiency of light capture and charge collection efficiency. CTNRAs not only have TiO_2_ nanorod arrays that are grown perpendicularly to the FTO substrate with a large surface area, in case to enhance the light harvesting efficiency, but also have CuPc layer which can provide a direct pathway for excited holes to improve the collection efficiency and water oxidation kinetics. Accordingly, the IPCE of CTNRAs can reach as high as 45%.

**Figure 5:**
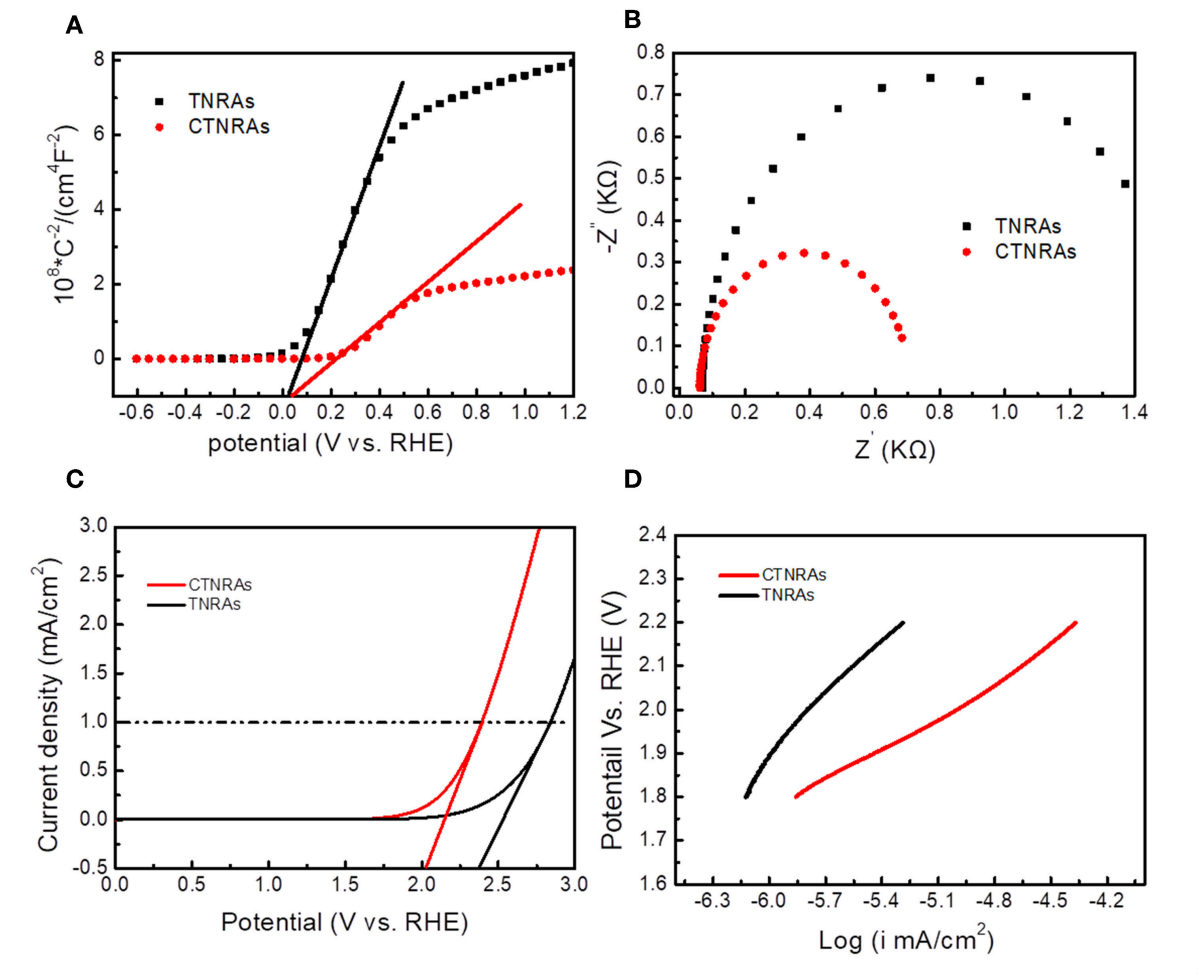
Mott-Schottky curves **(A)**; electrochemical impedance specra **(B)**; Polarization curves **(C)**, and Tafel plots **(D)** of pristine TNRAs and the CTNRAs with 60 s deposition.

In order to further verify the proposed mechanism, Mott-Schottky measurements were used to obtain flat band potential and the charge carrier density of the interface between semiconductors. The positive slope of CTNRAs and pristine TNRAs (Figure 5A) suggests the expected n-type semiconductor of TiO_2_ in the nanocomposites. For n-type semiconductor, flat band potential is consistent with the bottom of the conduction band. Pristine TNRAs and CTNRAs have the same flat band voltage, implying that the deposition of CuPc did not change the flat band position of TNRAs. From the results of Mott-Schottky measurements, we excluded the possibility of p-n junction formation between the interface of CuPc and TNRAs, which also confirmed by the IPCE measurements where no response was observed from the longer wavelength according to the absorption of CuPc.

In order to further understand the enhanced PEC performance, the inherent electronic properties of CTNRAs were characterized by measuring electrochemical impedance spectroscopy (EIS), the onset OER potential and linear Tafel plots. It is well-known that the larger over potential of TNRAs for water oxidation, presenting the sluggish kinetics, which can limits its wide application in PEC water splitting. The onset potential for OER of TNRAs and CTNRAs (Figure 5C) is 2.36, and 2.04 V, respectively, which indicate electrocatalytical activity in CTNRAs was greatly enhanced by the deposition of CuPc. Electrochemical impedance is an effective method for assessing the kinetic of electron transfer at the electrode-electrolyte interface. From Figure 5B, electrochemical impedance spectra show that pristine TNRAs has higher resistance than CTNRAs, indicating the loading of CuPc effectively promote the separation of electron and hole and enable high-speed electron transport for water splitting.

The Tafel plot depicts the relationship between the logarithm of the current density (ik) and the over potential (η), which is an important parameter for water spiltting electrocatalysts and can provide important information about electronic enhancement of electrocatalysts activity (Tong et al., 2014). Generally, the decrease in the slope of the curve represents an increased kinetics for PEC oxygen revolution. As shown in Figure 5D, the slope of the Tafel curve for TNRAs and CTNRAs is about 405, and 256 mV per decade, respectively. The result show CTNRAs has lower slop value than TNRAs, which can indicate TNRAs has a more sluggish kinetics than CTNRAs.

**Figure 6:**
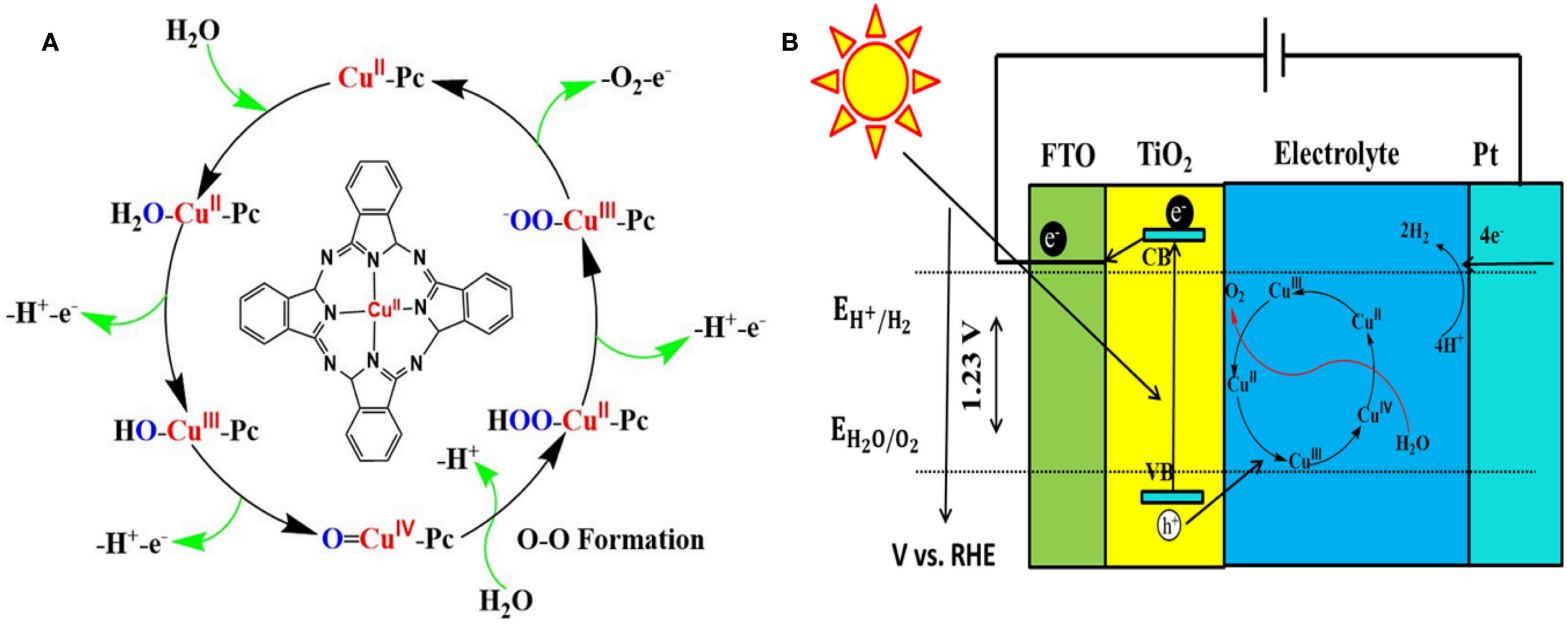
**(A)** Proposed water-oxidation mechanism of CuPc catalysis, **(B)** Schematic diagram for transfer and separation of photogenerated charges and holes in the CTNRAs heterostructure.

To validate the mechanism of PEC performance enhancement by CuPc deposition, we compared the cyclic voltamn spectra of TNRAs and CTNRAs, there exist two unconspicuous oxidation peaks located at about 1.3 and 1.8 V vs. RHE in the CV curve of CTNRAs, while there are not any oxidation peaks in the same range in the CV curve of TNRAs (Figure S13). Then the LSV curve of CuPc deposited on FTO with that of blank FTO, as shown in Figure S14A, the onset potential of CuPc is about 2.01 V vs. RHE, while the onset potential of blank FTO anode is about 2.29 V vs. RHE under the same conditions. Obvious cathodic shift of onset potential about 280 mV is observed, indicating that CuPc has acted as a co-catalyst for electrochemical water oxidation which is coincident with the results of Mott-Schottky. In order to further clarify the detailed process of water oxidation catalyzed by CuPc, the cycle voltammetry (CV) curves were measured and the results was shown in Figure S14B. Compared with the CV curve of blank FTO, there appeared two more oxidation peaks located at 1.31 and 1.69 V vs. RHE in the CV curve of CuPc, which could be attributed to the oxidation process of Cu^2+^ to Cu^3+^ and Cu^3+^ to Cu^4+^, respectively (Zhang et al., 2013). Based on the electrochemical analysis above, a possible mechanism for water-oxidation catalyzed by CuPc was proposed (Figure 6A). During the PEC process, a water molecule from solution firstly coordinated to Cu center of CuPc molecule anchored on the surface of the elelctrod to form hydrated copper phthalocyanine (Li et al., 2012). As follows, the resulting H_2_O=Cu^II^-Pc is oxidized through a PCET to yield a HO=Cu^III^-Pc, which can be further oxidized to a O=Cu^IV^-Pc by PCET. Next, O=Cu^IV^-Pc is attacked by H_2_O or OH^−^ from the electrolyte, correspondingly, and O-O bond can be generated. The process is in accordance with the water nucleophilic attack (WNA) mechanism. HOO-Cu^II^-Pc is an important intermediate in the peroxide bridge structure, which can be further oxidized to a ^−^OO=Cu^IV^-Pc by PCET, then releases oxygen molecules and the CuPc species restore to the initial state.

Based on the characterizations as mentioned earlier, a possible mechanism for the PEC performance improvement over CTNRAs is proposed as shown in Figure 6B. Under simulated sunlight irradiation, photo generated electrons are excited from the valence band (VB) of TiO_2_ to its conduction band (CB), leaving holes in VB of TiO_2_. Ultimately, electrons pass through the external circuit to the counter electrode and H^+^ were reduced to produce H_2_. Accordingly, the holes from VB of TiO_2_ were consumed by the process in which Cu^2+^ is oxidized to Cu^3+^ and then to Cu^4+^. In the process of water oxidation, the presence of CuPc derivated species is considered to be a fast redox mediator, which not only reduce the activation energy of PEC water oxidation, but promote effective charge separation as well.

## Conclusion

In conclusion, through a simple electro-induced assembly method π-conjugated copper phthalocyanine was successfully deposited on the surface of TiO_2_ nanorod arrays to form organic-inorganic hybrid nanostructures which were directly grown on the FTO substrate. The obtained hybrid nanostructures can be used as photoanode for PEC water splitting with enhanced performance and good stability. Electrodeposited copper phthalocyanine molecules were proved to act as a co-catalyst for PEC water oxidation rather than a p-type semiconductor to form p-n junction with TiO_2_. Detailed mechanism was also proposed based on detailed experiments and analysis. This design establishes a cost-effective surficial assembly strategy to fabricate PEC device with enhanced performance using functional π-conjugated molecules in the field of PEC water splitting, carbon dioxide reduction and other related energy-storing reactions.

## Author Contributions

YL designed experiments. MY carried out experiments. ZT, NL, and HZ analyzed experimental results. YaL, AZ, and SX analyzed sequencing data and gave helpful discussions to the conclusions. MY and YL wrote the manuscript.

### Conflict of Interest Statement

The authors declare that the research was conducted in the absence of any commercial or financial relationships that could be construed as a potential conflict of interest.
