# Supplementary material for: Assembly of Copper Phthalocyanine on TiO2 Nanorod Arrays as Co-catalyst for Enhanced Photoelectrochemical Water Splitting
Source: Front Chem. 2019 May 14;7:334. doi: 10.3389/fchem.2019.00334 (PMC6530342; doi:10.3389/fchem.2019.00334)
Supplement: Supplementary file 1 [file Data_Sheet_1.docx]

Supplementary Material

**Assembly of Copper Phthalocyanine on TiO_2_ Nanorod Arrays as Co-catalyst for Enhanced Photoelectrochemical Water Splitting**

**Yuangang Li ^1,2*^, Mengru Yang ^1^, Zimin Tian ^1^, Ningdan Luo ^1^, Yan Li ^2^, Haohao Zhang ^1^, Anning Zhou ^1,2^, Shanxin Xiong ^1,2*^**

^1^ College of Chemistry and Chemical Engineering, Xi’an University of Science and Technology, Xi’an, 710054, China

^2^ Key Laboratory of Coal Resources Exploration and Comprehensive Utilization, MLR., 710021

^3^Key Laboratory of Synthetic and Natural Functional Molecule Chemistry of the Ministry of Education, College of Chemistry and Materials science, Northwest University, Xi’an 710069, China

* Correspondence:
Corresponding Author: Yuangang Li
email: [Liyuangang@xust.edu.cn](mailto:Liyuangang@xust.edu.cn)


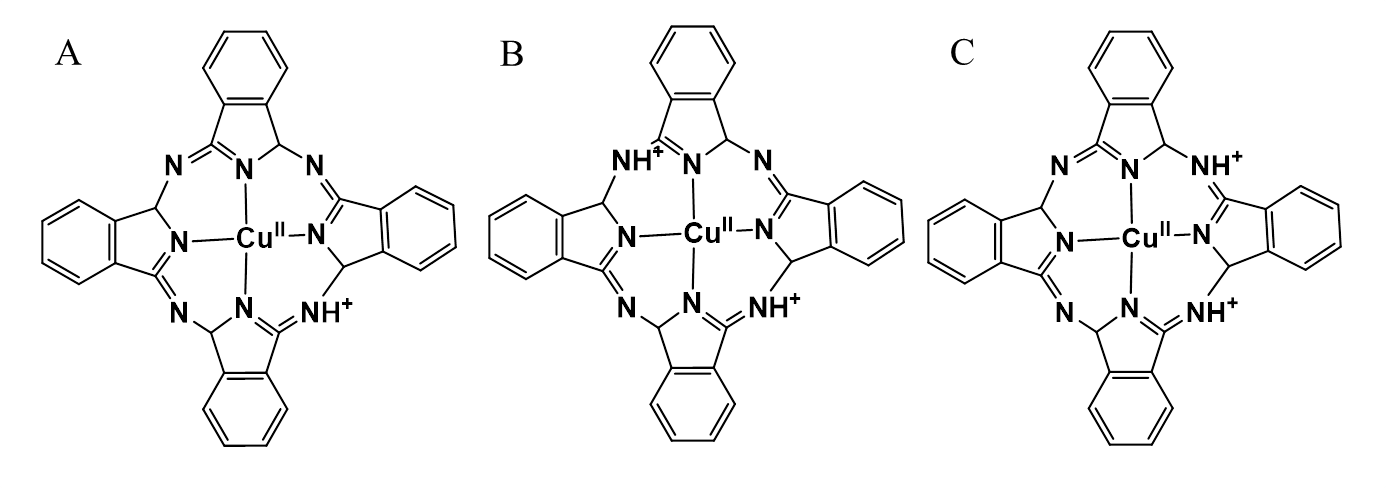


**Figure S1.** The molecular structure of protonated CuPc.


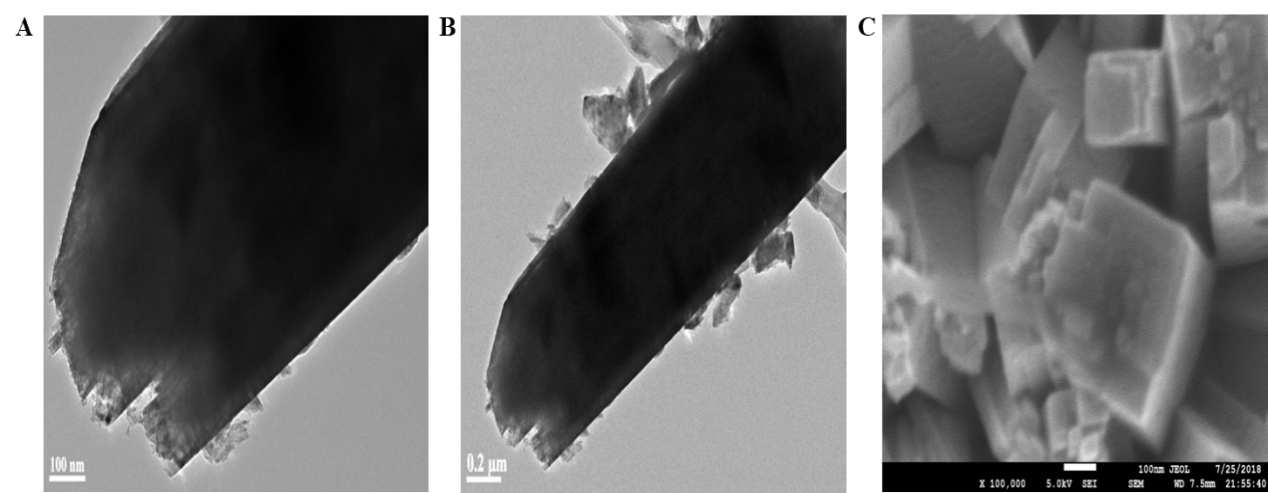


**Figure S2.** TEM images (A, B) and SEM image (C) of the TNRAs.


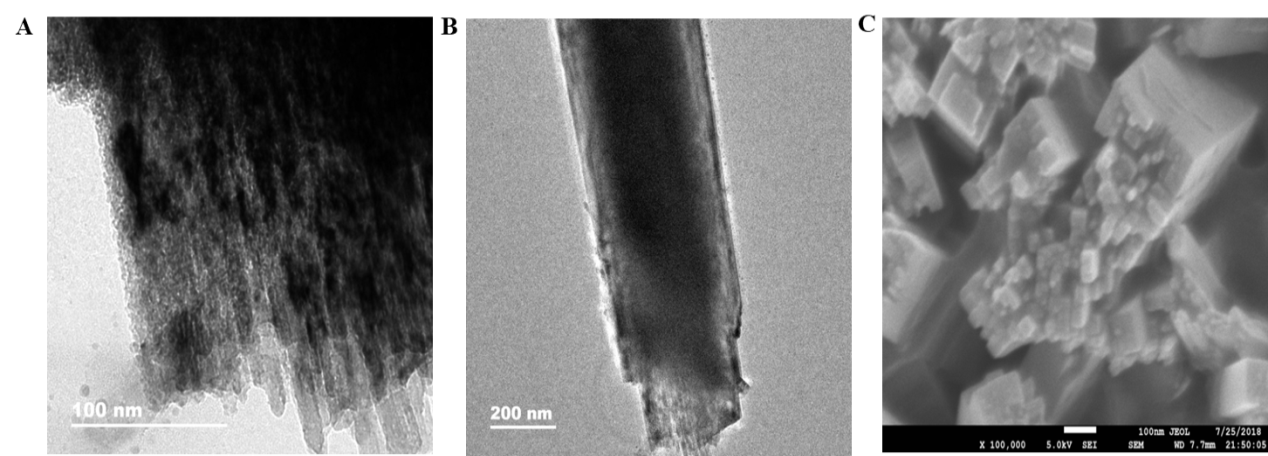


**Figure S3.** TEM images (A, B) and SEM image (C) of the CTNRAs.


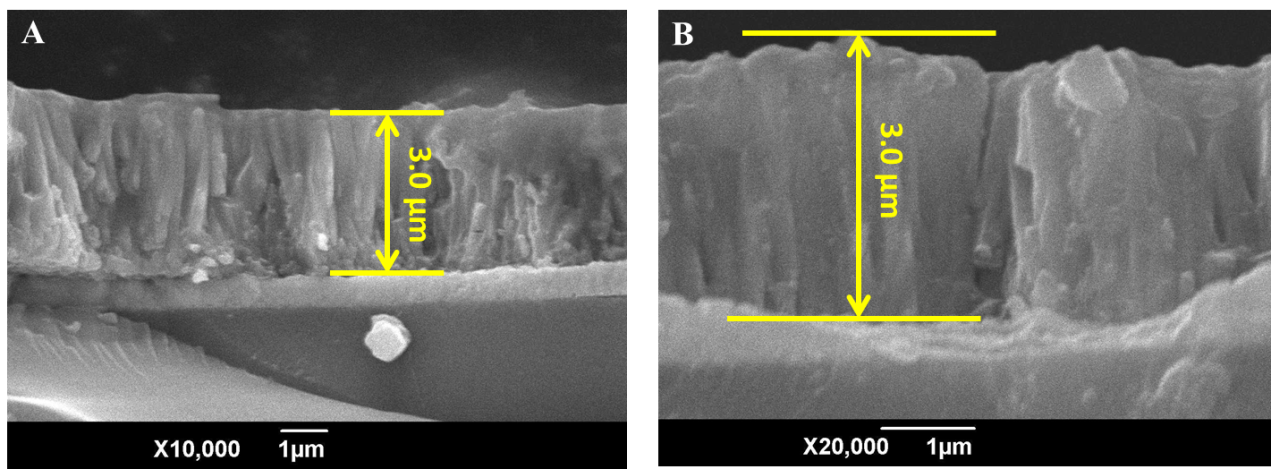


**Figure S4.** SEM side view of (A) TNRAs and (B) CTNRAs on FTO substrates.


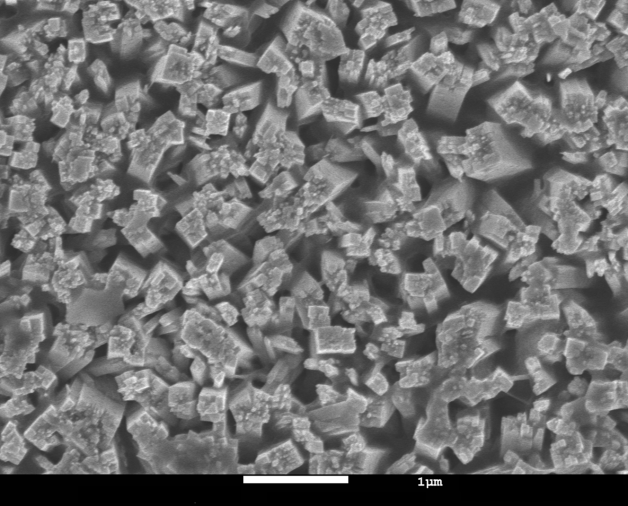


**Figure S5.** SEM of CTNRAs after 8 h PEC test.


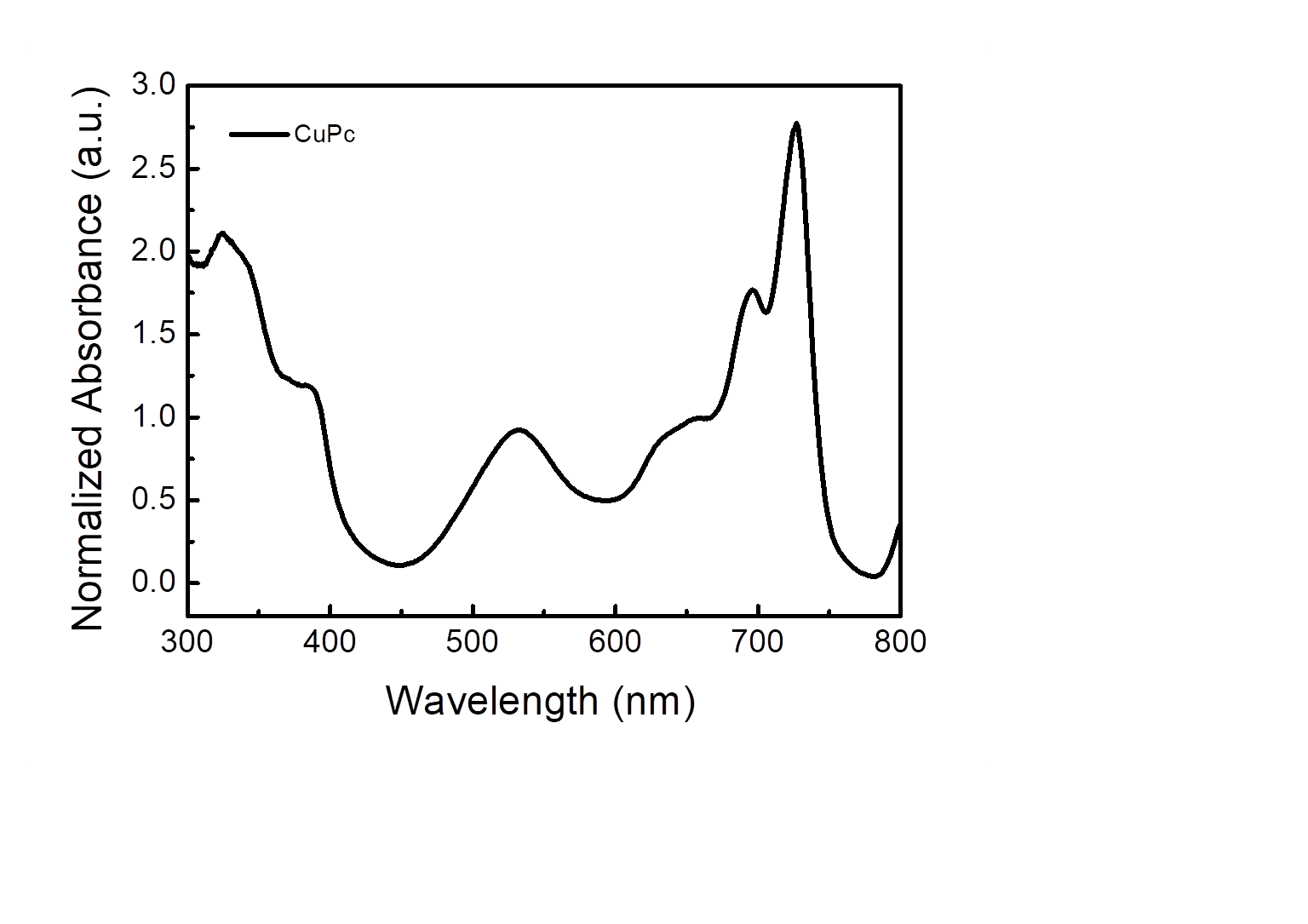


**Figure S6.** UV-vis absorption spectra of CuPc in electroplate solution.


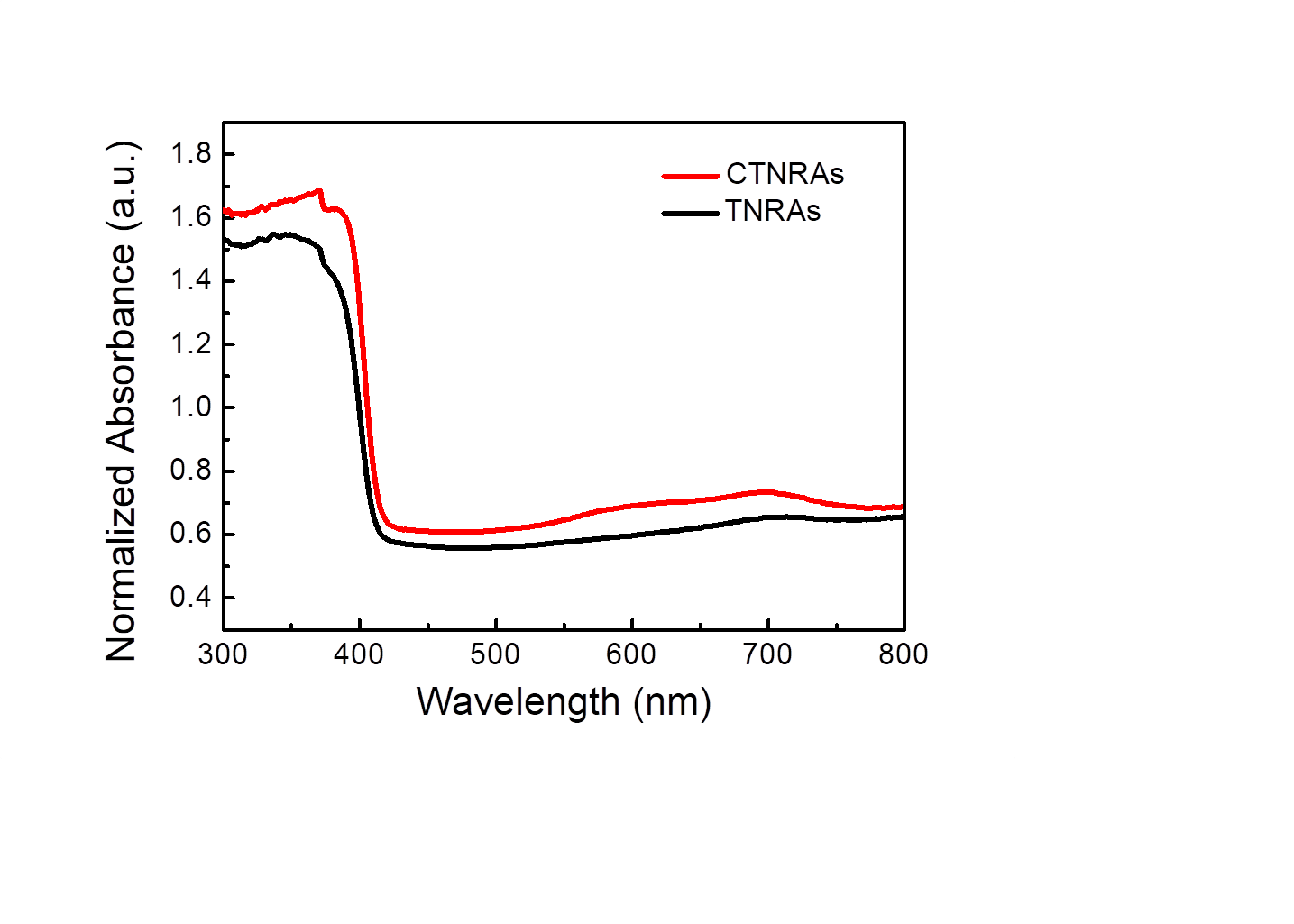


**Figure S7.** UV-vis diffuses reflectance spectra of TNRAs and CTNRAs.


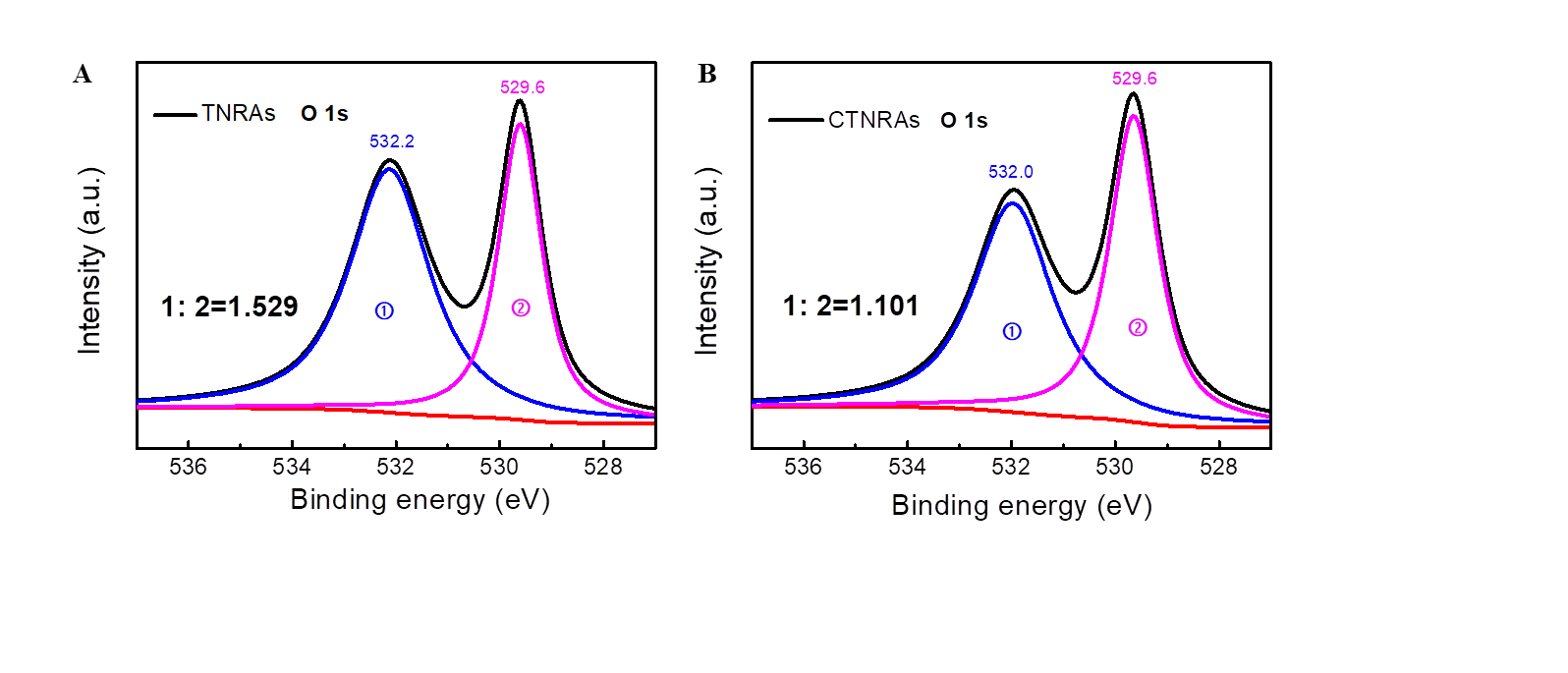


**Figure S8.** High-resolution XPS spectrum for O 1s of TNRAs (A) and CTNRAs (B).

**
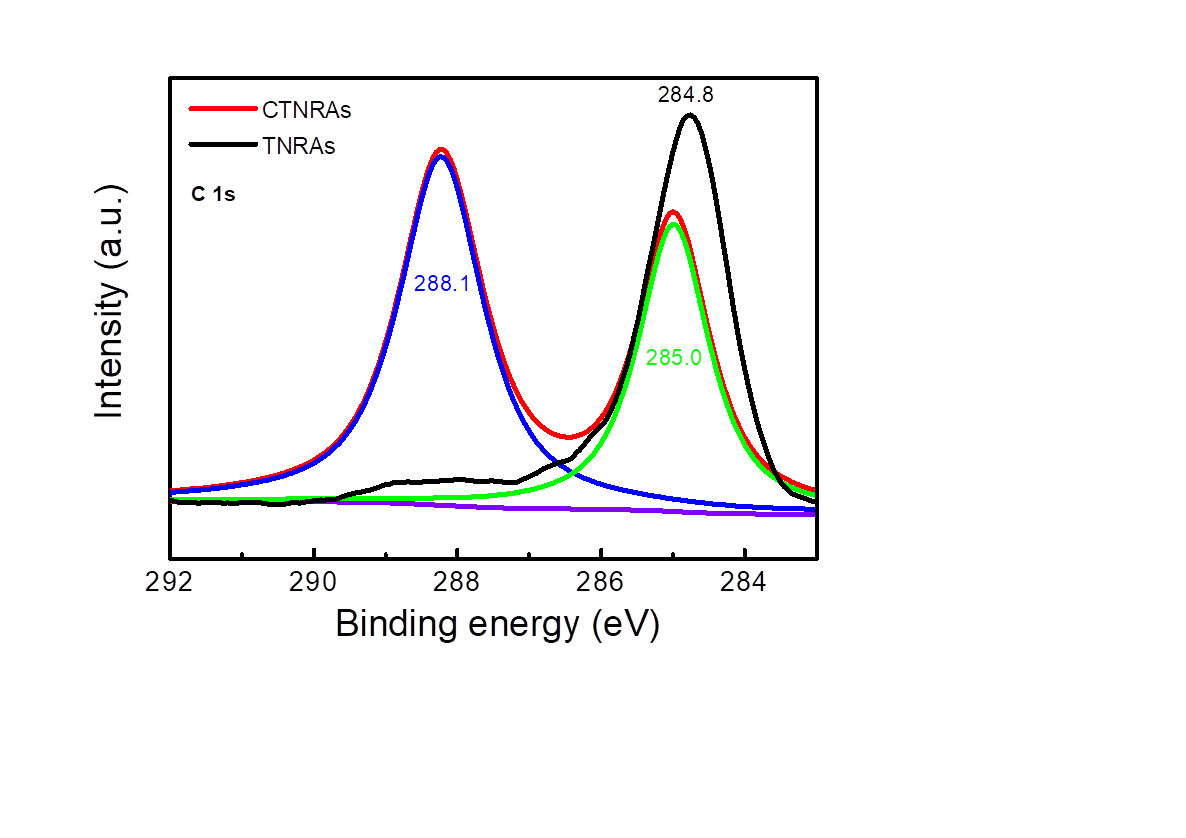
**

**Figure S9.** High-resolution XPS spectrum for C 1s of TNRAs and CTNRAs.


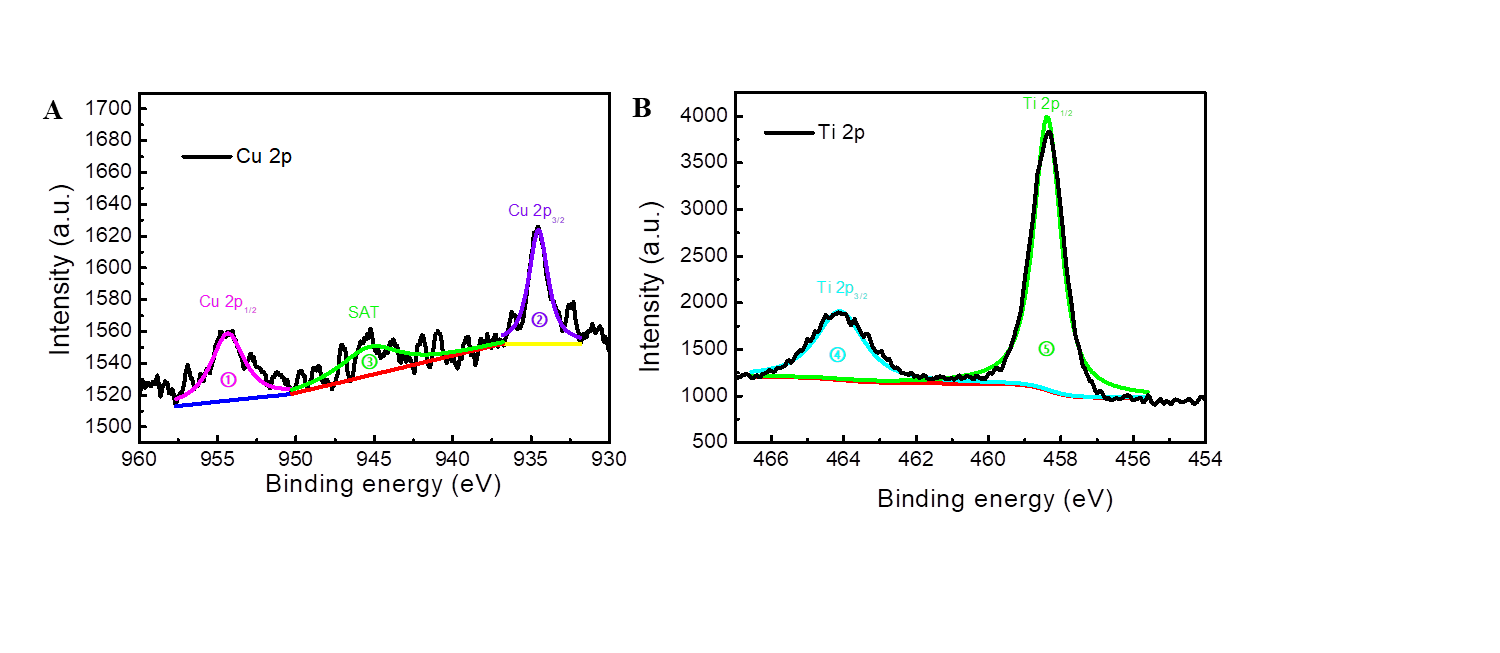


**Figure S10.** High-resolution XPS spectrum for Cu 2p (A) and Ti 2p (B) of CTNRAs.

Element ratio of Ti and Cu of TiO_2_@CuPc sample which electro-deposition time was 60 s could be

expressed concretely as：

$$\mathbf{Element ratio=}\frac{{\mathbf{S}_{\mathbf{Ti}}}_{\boldsymbol{2}\boldsymbol{p}}\boldsymbol{\div}\boldsymbol{SF}_{\boldsymbol{Ti}}}{\boldsymbol{S}_{\boldsymbol{Cu}_{\boldsymbol{2}\boldsymbol{p}}}\boldsymbol{\div}\boldsymbol{SF}_{\boldsymbol{Cu}}}\boldsymbol{\times100}\boldsymbol{\%}$$

Where S_Ti_**_2p_** and S_Cu_**_2p_** were the intergral area, the SF_Ti_ and SF_Cu_ were the sensitivity factor of Ti and Cu, respectively.$\frac{(S_{4}+S_{5})\div2.0980}{(S_{1}+S_{2}+S_{3})\div4.8710}=\frac{5558\div2.0980}{428\div4.8710}=30.15$, So the element ratio of Ti:Cu of best TiO_2_@CuPc sample was about 30:1.


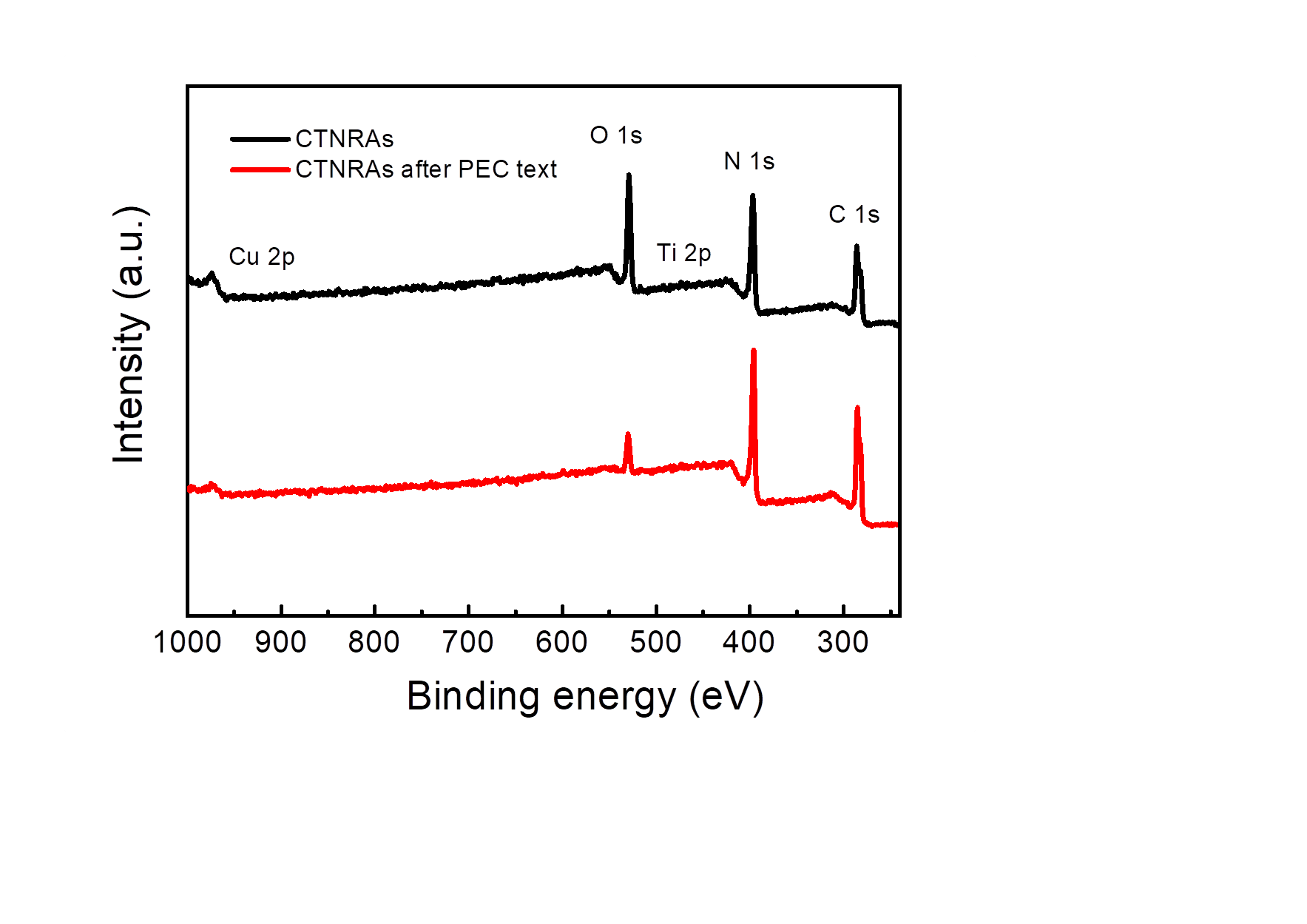


**Figure S11.** XPS of CTNRAs after 8 h PEC operation.


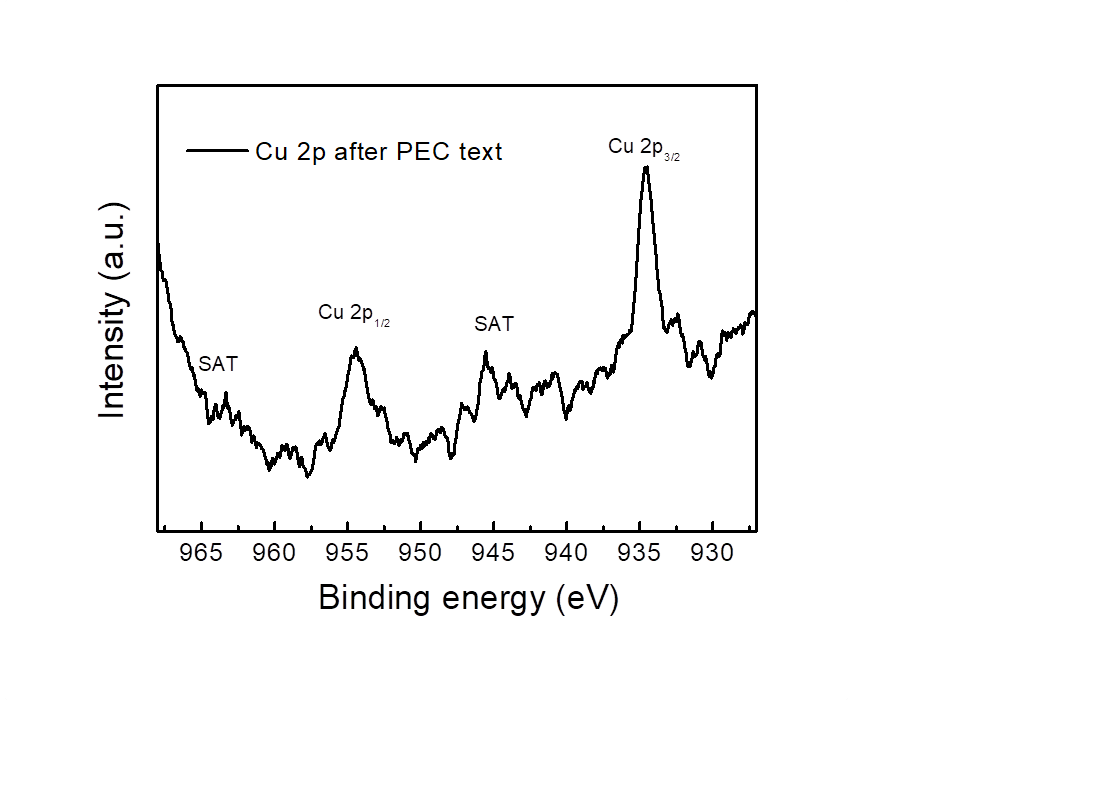


**Figure S12.** XPS of Cu 2p of CTNRAs after 8 h PEC operation.

**
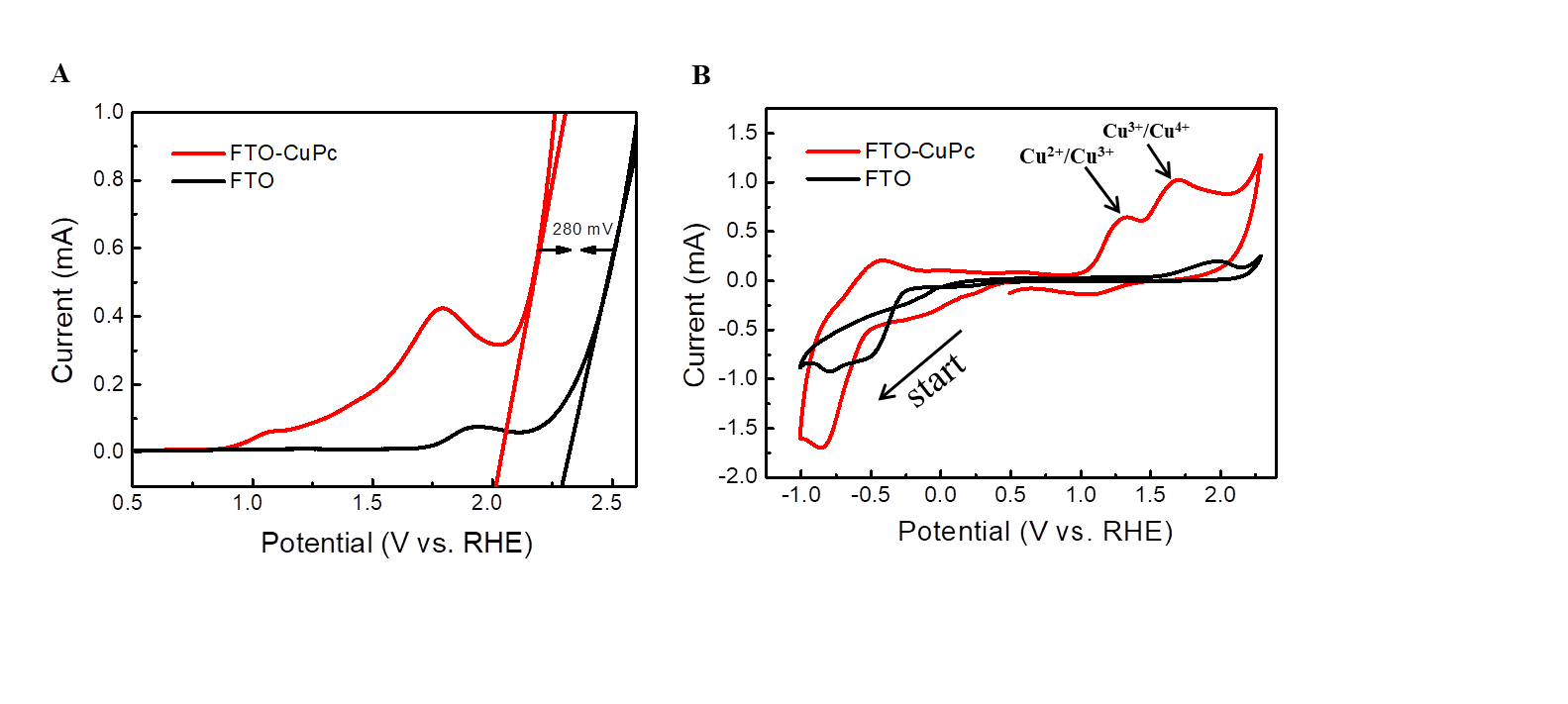
**

**Figure S13.** Photocurrent density-potential curves (A) and cyclic voltamn spectra (B) of CuPc.


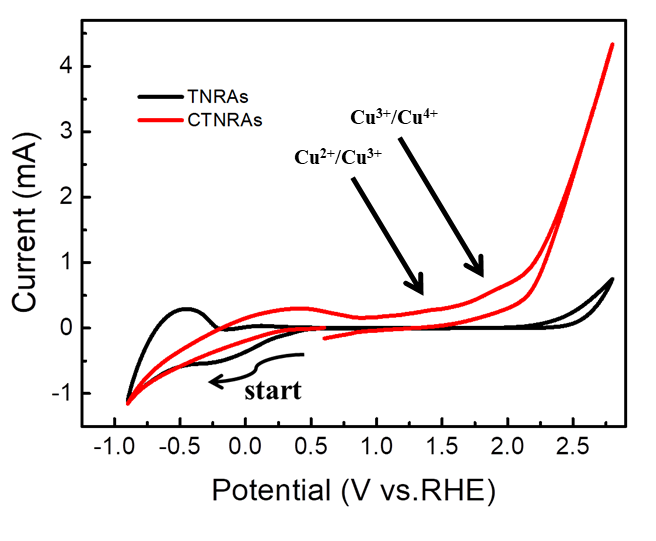


**Figure S14.** Cyclic voltamn spectra of TNRAs and CTNRAs.

**
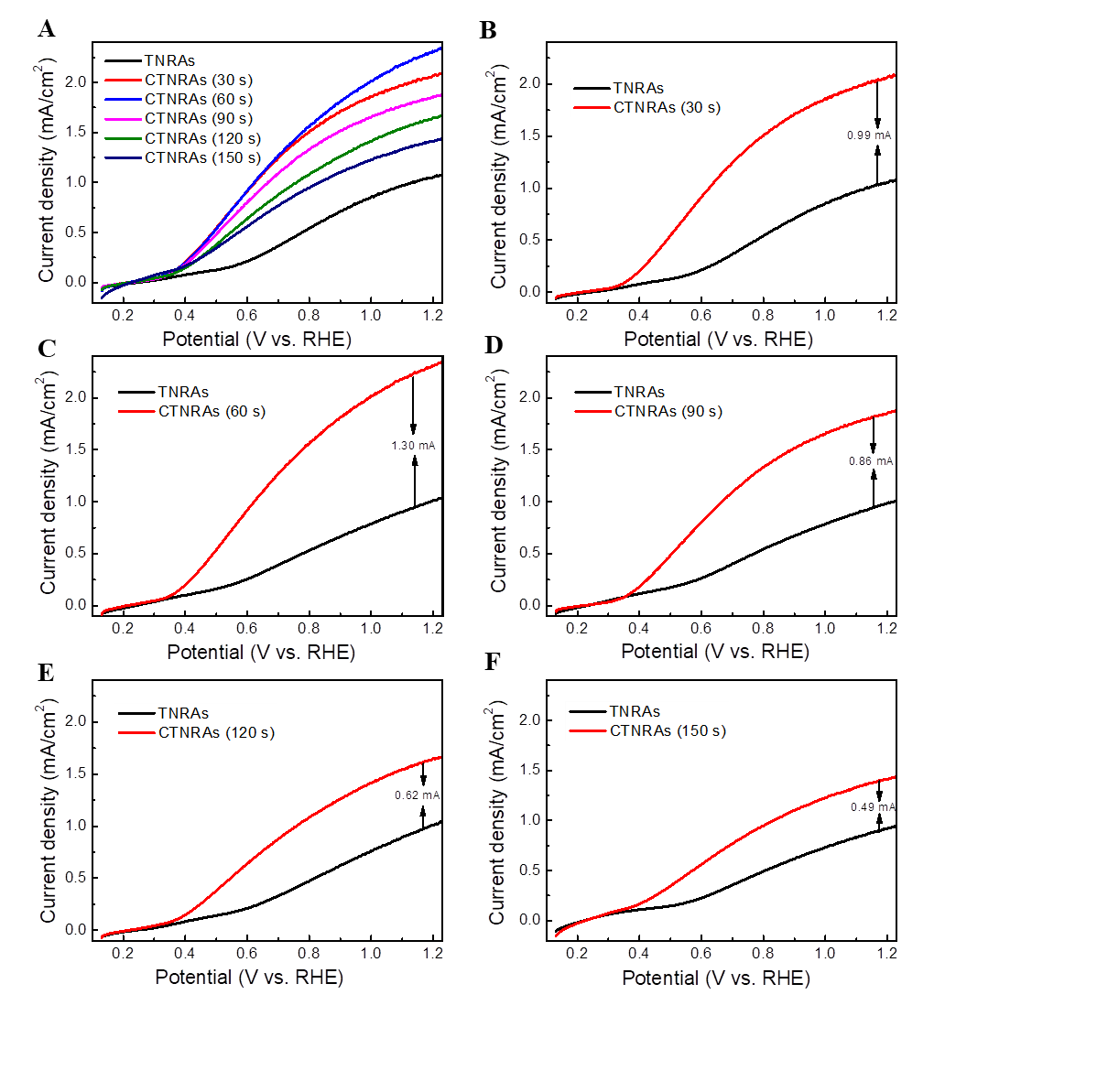
**

**Figure S15.** (A) Photocurrent density-potential curves of pure TNRAs and the CTNRAs by electro-deposition with different time, under AM 1.5G (100 mW/cm^2^); the spectra of electro-deposition time

is 30 s (B), 60 s (C), 90 s (D), 120 s (E) and 150 s (F).

**
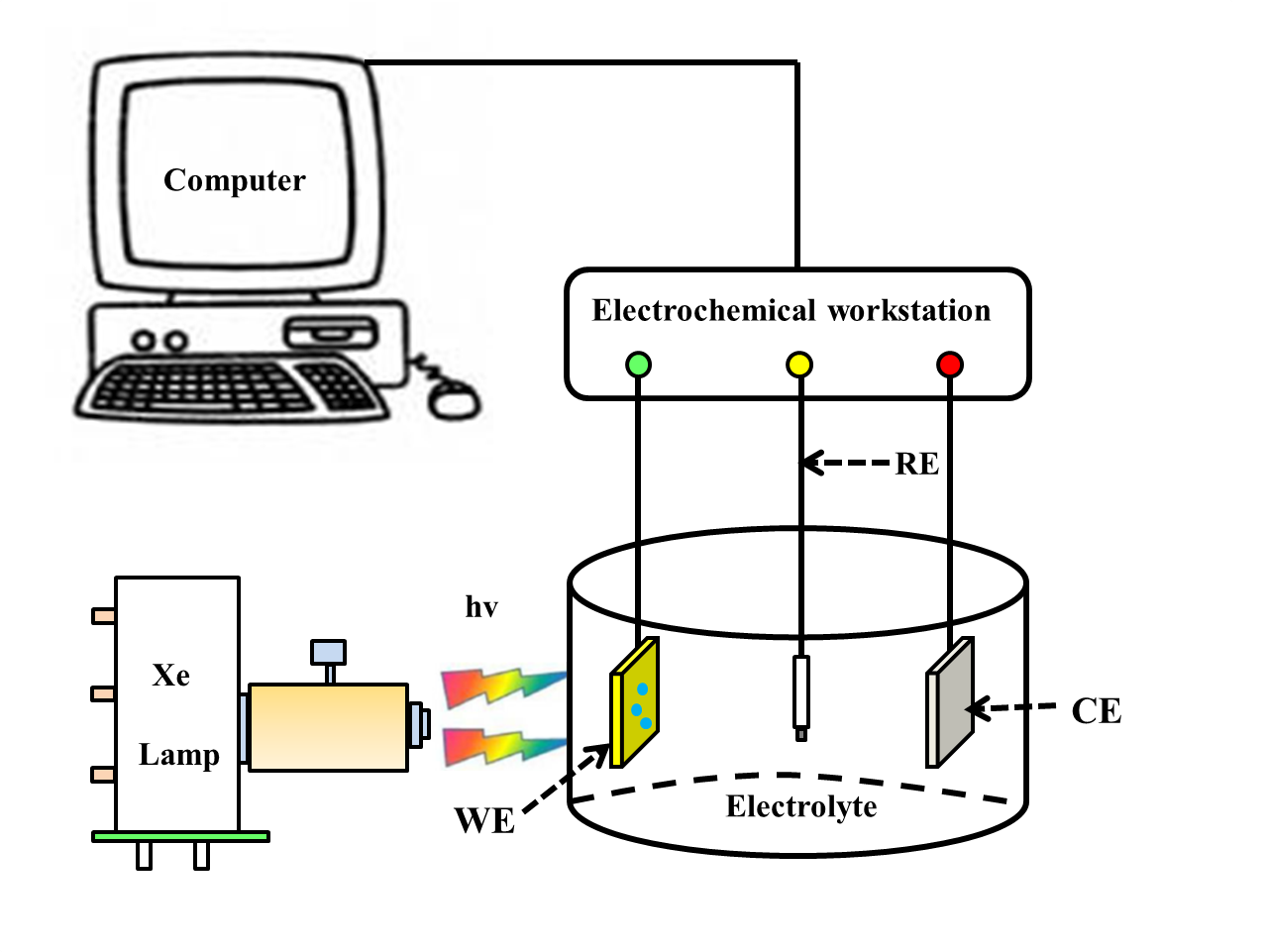
**

**Figure S16.** A Schematic illustration for the geometry and design of PEC reactor.
